# NLRP3 activation induces ASC-dependent programmed necrotic cell death, which leads to neutrophilic inflammation

**DOI:** 10.1038/cddis.2013.169

**Published:** 2013-05-23

**Authors:** T Satoh, N Kambe, H Matsue

**Affiliations:** 1 Department of Dermatology, Chiba University Graduate School of Medicine, Chiba, Japan

**Keywords:** NLRP3, inflammasome, CAPS, programmed necrotic cell death, IL-1*β*, neutrophil

## Abstract

NLR family pyrin domain containing 3 (NLRP3) is a cytoplasmic pattern recognition receptor that regulates innate immune responses by forming a protein complex, the inflammasome. It leads to production of proinflammatory cytokine productions such as interleukin 1*β* (IL-1*β*). We and others demonstrated that an induction of activated NLRP3 also induced cell death. However, little is known about the characteristics and mechanisms of the cell death and its involvement in the pathogenesis of inflammatory conditions. In this study, we established cell lines in which NLRP3 was induced by doxycycline using a tetracycline-inducible expression (Tet-on) system. Using this system, the expression of NLRP3 mutants in cryopyrin-associated periodic syndrome (CAPS) patients was sufficient for the induction of necrotic cell death without lipopolysaccharide stimulation or generation of mature IL-1*β*. We also found that CA074-Me, a cathepsin B inhibitor, blocked cell death before oligomerization of apoptosis-associated speck-like protein containing a CARD (ASC), whereas Z-VAD-fmk, a pan-caspase inhibitor, blocked the cell death after the oligomerization. Silencing of the ASC gene (*Pycard*) by small hairpin RNA treatment inhibited the NLRP3 mutant-induced cell death, but silencing of the caspase-1 gene (*Casp1*) did not. Taken together, these results indicated that ASC was indispensable for NLRP3-mediated programmed necrotic cell death, and that this type of cell death was distinct from ‘pyroptosis', which requires caspase-1. Finally, we demonstrated in an *in vivo* model that the programmed necrotic cell death induced by activated NLRP3 could cause neutrophil infiltration, indicating a possible role of cell death in neutrophil infiltration of skin lesions in CAPS patients.

NLR family pyrin domain containing 3 (NLRP3) is a cytoplasmic protein belonging to the NOD-like receptor (NLR) family.^1^ This family is characterized by pattern recognition receptors, and senses microbial molecules and danger signals in the cytoplasm of cells. NLRP3 and apoptosis-associated speck-like protein containing a CARD (ASC) are components of the inflammasome, a multiprotein complex required for caspase-1 activation and interleukin 1*β* (IL-1*β*) production. Formation of the NLRP3-inflammasome requires two signals. The first signal (also called the priming step) consists of microbial molecules or endogenous cytokines and is required for the upregulation of NLRP3 itself and the production of targeting cytokines by the inflammasome, including the pro-form of IL-1*β* (pro-IL-1*β*). The second signal is provided by extracellular ATP, pore-forming toxins or crystals. These signals activate NLRP3, leading to NLRP3 oligomerization followed by ASC oligomerization, and finally the formation of the NLRP3-inflammasome. Then, the inflammasome induces self-cleavage of pro-caspase-1 into the active form of caspase-1 that cleaves pro-IL-1*β* into the biologically active p17 form of IL-1*β* (mature IL-1*β*).^1^

The mammalian NLR family is structurally related to plant resistance (R) proteins.^2^ R proteins detect pathogen-effector or avirulence proteins, leading to production of antimicrobial proteins and programmed cell death that is localized at infection sites.^3^ Certain bacterial effectors have evolved the ability to suppress the programmed cell-death responses, allowing pathogen growth and disease in plants, suggesting the importance of programmed cell death in plants' innate immunity.^4^ Whereas a large number of recent publications have greatly increased our knowledge of the mechanisms involved in production and processing of IL-1*β* by the NLRP3-inflammasome, we are only beginning to understand the mechanisms of cell death caused by NLRP3 activation.

*NLRP3* (the gene that encodes cryopyrin) was originally identified as the gene responsible for cryopyrin-associated periodic syndrome (CAPS) in which neutrophilic urticarial rash is the most common symptom.^5^ CAPS is caused by gain-of-function mutations in *NLRP3* that cause constitutive activation of NLRP3 in the absence of second signals and secretion of IL-1*β*. Three independent groups, including this laboratory, have reported that monocytes isolated from CAPS patients were selectively induced to undergo cell death upon lipopolysaccharide (LPS) treatment,^6, 7, 8^ accompanied by mutant NLRP3 expression. Of potential interest are patients with NLRP3 mutation with somatic mosaicism. Monocyte cell death after LPS treatment was only observed in the cells bearing mutant NLRP3 but not in those without mutation.^6, 7, 8^ Moreover, we previously reported that the transient expressions of CAPS-associated mutant NLRP3 caused necrotic cell death.^9^ The observation that the necrosis was inhibited by cathepsin B inhibitor (which also blocks programmed cell death caused by R protein in plants^10^), suggested that it was indeed programmed cell death.

Caution is required in concluding that NLRP3 activation leads to programmed cell death. This is because LPS treatment induces an array of gene products including IL-6, IL-8, and tumor necrosis factor-*α* in addition to NLRP3 and pro-IL-1*β*. Thus, it is possible that LPS-induced factors could participate in cell death. In addition, the transfection methodology (i.e., lipid-mediated gene delivery) itself has been shown to activate the inflammasome.^11, 12, 13, 14^ Moreover, second signals alone are known to induce cell damage or cell death.^15, 16^ Thus, to conduct studies in an unambiguous manner, we generated cell lines in which NLRP3 was induced upon doxycycline treatment using a tetracycline-inducible expression (Tet-on) system, eliminating the need for LPS treatment or exogenous DNA transfection. In addition, we utilized CAPS-associated mutant NLRP3 to avoid the damage associated with second signals.

Using the Tet-on system, we demonstrated in this study that the expression of CAPS-associated NLRP3 mutants alone was sufficient for necrotic cell death without LPS stimulation. On the basis of the observation that the distribution pattern of ASC in the cytoplasm was quite different after the treatment of two inhibitors, we demonstrated that CA074-Me inhibited the cell death before ASC oligomerization, whereas Z-VAD-fmk inhibited the cell death after ASC oligomerization. We also showed that NLRP3-mediated cell death can recruit neutrophils even in the absence of IL-1*β.* This characteristic cell death-mediated neutrophil-rich inflammation has wider significance because it is mediated by NLRP3, which responses to not only pathogens but also to danger-associated signals.

## Results

### Cell death induced by NLRP3 activation was dispensable for IL-1*β* expression

**Figure 1:**
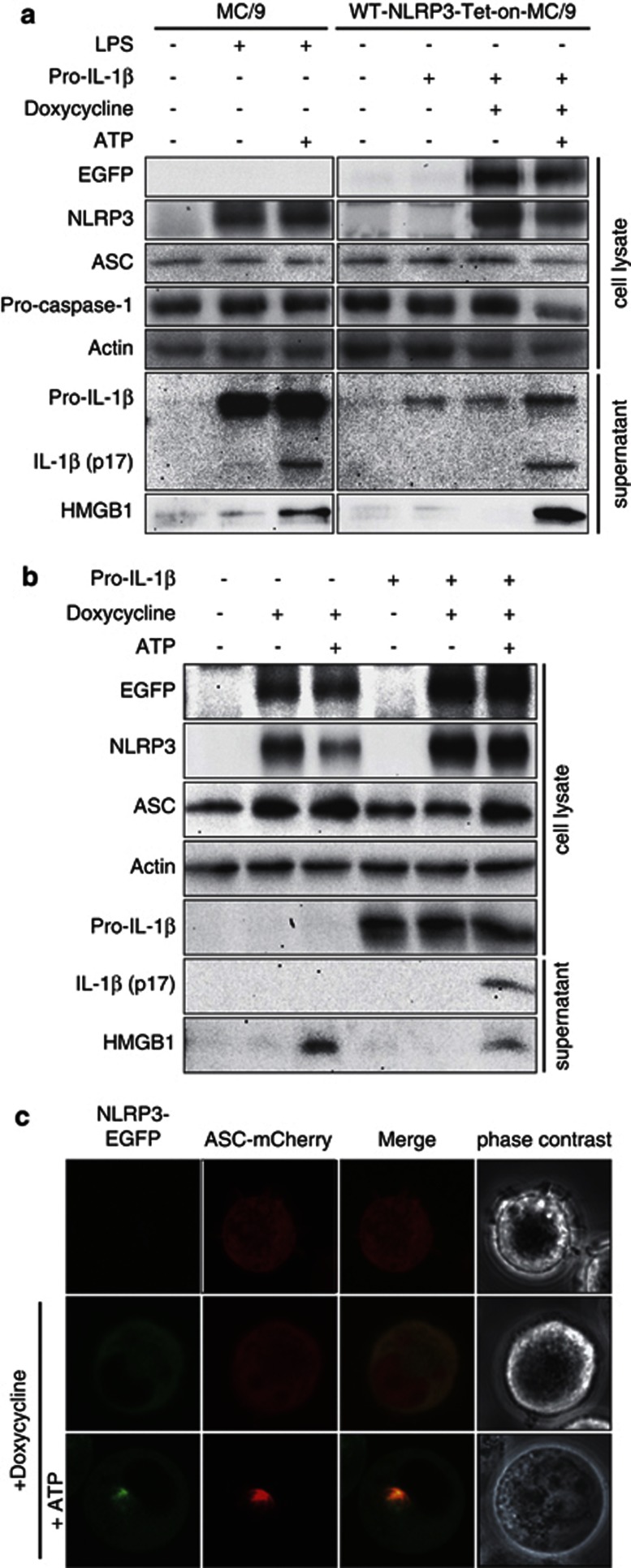
The first signals for the NLRP3-inflammasome can be replaced by expressions of NLRP3 and pro-IL-1*β*. (**a** and **b**) MC/9 cells (1 × 10^6^) were pretreated with 1 *μ*g/ml LPS for 2 h and stimulated with 5 mM ATP for 45 min. WT-NLRP3-Tet-on-MC/9 cells were transfected with the pro-IL-1*β*-pMX-IP retroviral vector and incubated for 48 h before ATP stimulation. WT-NLRP3-Tet-on-MC/9 cells (1 × 10^6^) were treated with 1 *μ*g/ml doxycycline for 12 h before ATP stimulation to induce the expression of WT-NLRP3. Cells and supernatants obtained from experiments using MC/9 and WT-NLRP3-Tet-on-MC/9 were harvested 45 min after ATP stimulation, except supernatants for HMHB1 immunoblotting that were harvested 12 h after ATP stimulation. (**c**) WT-NLRP3-Tet-on-MC/9 cells stably expressing ASC-mCherry were pretreated with 1 *μ*g/ml doxycycline for 12 h and stimulated with 5 mM ATP. ATP stimulation induced the co-localization of NLRP3 and ASC speckles, and induced cell swelling, leading to necrotic cell death

NLRP3-inflammasome formation and mature IL-1*β* release requires two distinct signals. The most common examples are LPS as the first signal and ATP as the second signal. The mast cell line MC/9 stably expressed ASC and pro-caspase-1 under unstimulated conditions (Figure 1a, left panels). LPS treatment induced NLRP3 and pro-IL-1*β* expression, but mature IL-1*β* was observed only after ATP treatment. These observations were confirmed with the monocyte cell line J774A.1 (Supplementary Figure S1a).

In order to express NLRP3 without the possible influence arising from a first signal, we established a method for artificial induction of NLRP3 with the Tet-on system. Transfection of an expression vector coding pro-IL-1*β* into MC/9 cells induced the expression of pro-IL-1*β* without expressing NLRP3 (Figure 1a, right panels), and doxycycline treatment induced the expression of wild type (WT)-NLRP3 that was tagged with EGFP (WT-NLRP3-Tet-on-MC/9). The expression of both pro-IL-1*β* and WT-NLRP3 were insufficient to release mature IL-1*β* and further ATP stimulation was necessary. These data indicated that the artificial gene induction system obviated the need for LPS as a first signal.

Following NLRP3 induction and ATP stimulation, we observed the release of high-mobility group box 1 (HMGB1), as well as mature IL-1*β* (Figure 1a). HMGB1 is a strong proinflammatory factor and normally maintained within the nucleus but released from cells undergoing necrosis.^17^ Those results suggested that NLRP3 activation accompanied with mature IL-1*β* release could lead to necrotic cell death. However, we noted that even without expression of pro-IL-1*β* or cleavage of mature IL-1*β*, WT-NLRP3 induction and subsequent ATP stimulation induced HMGB1 (Figure 1b) and LDH release (Supplementary Figure S1b). This indicated that mature IL-1*β* was not required for NLRP3-mediated necrotic cell death.

Microscopic observation revealed that doxycycline treatment induced EGFP expression, indicating the induction of NLRP3 in the cytoplasm of WT-NLRP3-Tet-on-MC/9 cells (Supplementary Figure S1c). ATP stimulation induced an EGFP speckling in the cytoplasm (Supplementary Figure S1c). When WT-NLRP3-Tet-on-MC/9 cells co-expressed mCherry-tagged ASC, we observed red fluorescence, indicating that ASC was widely distributed in the cell (Figure 1c). ATP stimulation induced speckle formation of both ASC and NLRP3, and these speckles were co-localized (Figure 1c). These experiments were performed without pro-IL-1β expression, suggesting formation of the NLRP3-inflammasome even in the absence of pro-IL-1β. Cell swelling (Figure 1c) followed by membrane rupture was observed after ASC speckle formation.

### Induction of CAPS-associated NLRP3 mutants was sufficient for cell death

**Figure 2:**
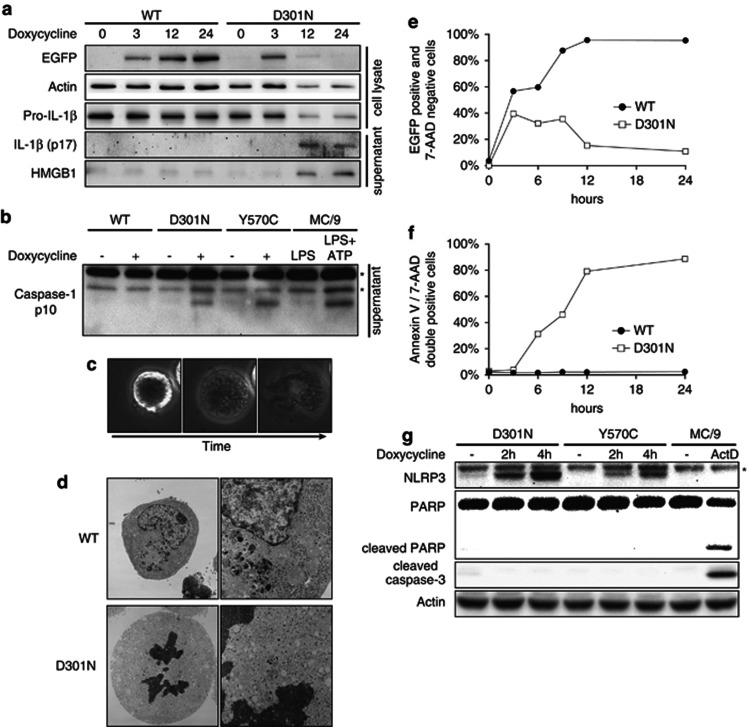
CAPS-associated mutant NLRP3-induced necrotic cell death in the absence of pro-IL-1*β*. (**a**) WT and D301N-NLRP3-Tet-on-MC/9 cells were transfected with the pro-IL-1*β*-pMX-IP retroviral vector and incubated for 48 h. NLRP3-Tet-on-MC/9 cells (1 × 10^6^) were treated with 1 *μ*g/ml doxycycline. Cells and supernatants were harvested for western blotting at the indicated time points after doxycycline treatment. (**b**) WT, D301N and Y570C-NLRP3-Tet-on-MC/9 cells (1 × 10^6^) were stimulated with 1 *μ*g/ml doxycycline for 16 h. MC/9 cells were pretreated with 0.5 *μ*g/ml LPS for 2 h and stimulated with 5 mM ATP for 45 min. The supernatants were harvested for immunoblotting of caspase-1. The indicate non-specific band. (**c**) Confocal scanning image of cell death caused by the expression of CAPS-associated mutant NLRP3, taken 6 h after doxycycline treatment. Scanning was performed at 1-min intervals with an Olympus FV10i. (**d**) Upper panels: electron microscopy of WT-NLRP3-Tet-on-MC/9 cells 10 h after doxycycline treatment. Lower panels: electron microscopy of D301N-NLRP3-Tet-on-MC/9 cells 10 h after doxycycline treatment. (**e** and **f**) WT and D301N-NLRP3-Tet-on-MC/9 cells were treated with 1 *μ*g/ml doxycycline, and the signal intensities of EGFP and Alexa647-annexin-V/ 7-AAD staining were analyzed by flow cytometry. MC/9 cells were incubated with 20 *μ*g/ml nigericin for 6 h as a necrosis control. (**g**) CAPS-associated mutant NLRP3-Tet-on-MC/9 cells (1 × 10^6^) were treated with 1 *μ*g/ml doxycycline for the indicated time and the cells were harvested for immunoblotting of NLRP3, PARP, cleaved caspase-3 and actin. The asterisk indicates non-specific band. MC/9 cells were incubated with 100 nM actinomycin D for 8 h as an apoptosis control

Even though ATP stimulation alone did not induce HMGB1 release (Supplementary Figure S2a), ATP is known to induce cell damage.^15, 16^ Thus, we used CAPS-associated, spontaneously active NLRP3 mutants^18^ to avoid ATP-induced cell damage, enabling us to examine whether or not the necrotic cell death observed was the consequence of inflammasome formation. The induction of mouse NLRP3 mutants (R258W, D301N and Y570C), corresponding to the major human CAPS-associated-mutations (R260W, D303N and Y570C, respectively),^19^ by the Tet-on system in the presence of pro-IL-1*β* resulted in the release of mature IL-1*β* (Figure 2a and Supplementary Figure S2b) and caspase-1 activation (Figure 2b) even without a second signal after doxycycline treatment.

Unlike WT-NLRP3, expression of mutant NLRP3 in the Tet-on system showed HMGB1 release in the absence of ATP stimulation (Figure 2a and Supplementary Figure S2b), indicating that HMGB1 release was not the consequence of ATP stimulation but that of activated NLRP3 expression. HMGB1 release was also observed in the absence of pro-IL-1*β* without ATP stimulation (Supplementary Figure S2b). This indicates that pro-IL-1*β* was necessary for mature IL-1*β* release but not for NLRP3-related cell death. The same results were obtained from macrophage cell line J774A.1. The induction of CAPS-associated NLRP3 mutants produced mature IL-1*β* without a second signal (Supplementary Figure S2c) and resulted in HMGB1 release even without IL-1*β* cleavage (Supplementary Figure S2d).

### Cell death induced by CAPS-associated NLRP3 mutants was necrotic

Microscope observation of NLRP3-Tet-on-MC/9 cells showed that mutant NLRP3 expression induced rapid cell swelling, cell membrane rupture and release of cell contents outside the cells (Figure 2c). Rapid swelling and membrane rupture were also observed in mutant NLRP3-Tet-on-J774A.1 cells (Supplementary Figure S2e), indicating that cell death with necrotic features was not specific to MC/9 cells. Electron microscopy revealed loss of the nuclear membrane cavity and fusion of chromatin with the cytosol, as well as obscured structures of cytosolic organelles in mutant NLRP3-expressing cells (Figure 2d).

The increases of EGFP intensity (an indicator of the level of EGFP-tagged NLRP3 expression) were similar between WT and D301N-NLRP3-Tet-on-MC/9 cells for the first 3 h after doxycycline treatment (Figures 2a and e and Supplementary Figure S3a). Cells expressing WT-NLRP3 continued the increase in EGFP intensities (Figures 2a and e and Supplementary Figure S3a), whereas cells expressing D301N stopped the increase of EGFP intensities after 6–24 h and became 7-amino-actinomycin (7-AAD) positive (Figures 2a and e and Supplementary Figure S3a). Fluorescent dyes such as 7-AAD and TOTO-3 iodide (TOTO-3) penetrate dead or damaged cells and label DNA. Together with annexin-V (which bears a high affinity for the phosphatidylserine that is externalized in early stages of apoptosis), 7-AAD can identify cell status. By expressing the CAPS-associated mutant NLRP3 (D301N), the annexin-V-/7-AAD-double negative viable MC/9 cells shifted directly to annexin-V-/7-AAD-double positive. This transition was similar to nigericin-treated necrotic cells that did not pass through an annexin-V-positive/7-AAD-negative apoptotic stage (Figure 2f and Supplementary Figure S3b), whereas cells expressing WT-NLRP3 remained viable. This result indicates that necrotic cell death was induced following the expression of mutant NLRP3.

Note further that none of the cells expressing mutant NLRP3 showed cleavage of either caspase-3 or PARP, both of which were observed in apoptotic cells treated with actinomycin D (Figure 2g). This observation also indicated that NLRP3-mediated cell death was not apoptotic. The same results were obtained in J774A.1 cells (Supplementary Figure S3c).

### Loss of viability mediated by CAPS-associated NLRP3 mutants was programmed cell death

**Figure 3:**
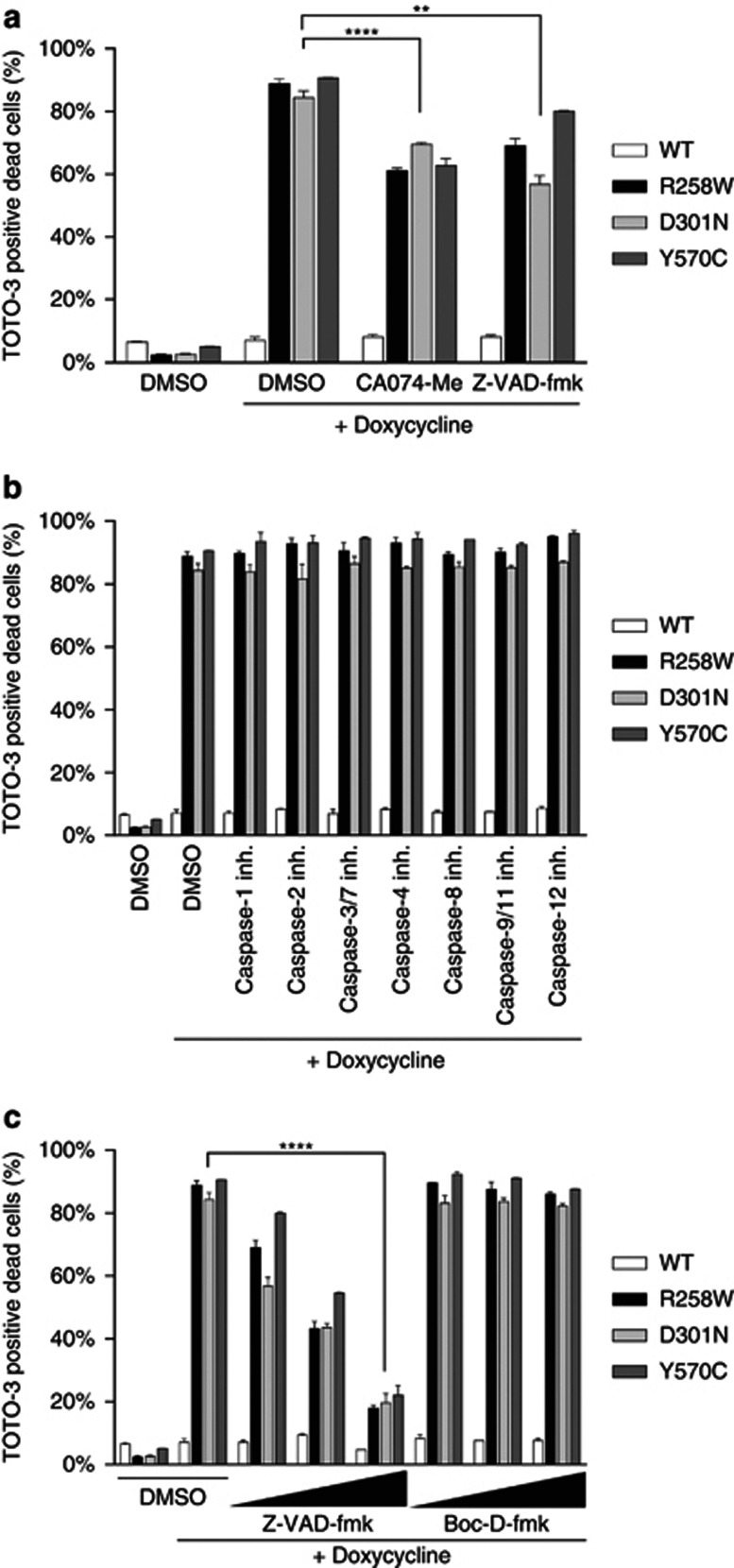
Cell death induced by CAPS-associated NLRP3 mutants was programmed cell death. (**a** and **b**) WT and mutant NLRP3-Tet-On-MC/9 cells were treated with 1 *μ*g/ml doxycycline for 12 h in addition to cathepsin B inhibitor CA074-Me (20 *μ*M), pan-caspase inhibitor Z-VAD-fmk (10 *μ*M), caspase-1 inhibitor Z-YVAD-cho (10 *μ*M), caspase-2 inhibitor, Z-VDVAD-fmk (10 *μ*M), caspase-3/7 inhibitor Z-DEVD-fmk (10 *μ*M), caspase-4 inhibitor Z-LEVD-fmk (10 *μ*M), caspase-8 inhibitor Z-IETD-fmk (10 *μ*M), caspase-9/11 inhibitor Z-LEHD-fmk (10 *μ*M) or caspase-12 inhibitor Z-ATAD-fmk (10 *μ*M). Cells were stained with 100 nM TOTO-3 for 5 min and analyzed by flow cytometry. (**c**) WT and mutant NLRP3-Tet-on-MC/9 cells were treated with 1 *μ*g/ml doxycycline for 12 h in addition to pan-caspase inhibitor Z-VAD-fmk or Boc-D-fmk (10/20/40 *μ*M). **, *P*<0.01; and ****, *P*<0.0001

In contrast to accidental cell death, programmed cell death is inhibited when its signaling pathways are blocked. Thus, we examined if necrotic cell death induced by mutant NLRP3 could be blocked with inhibitors. We previously reported that cell death induced by LPS treatment of monocytes isolated from CAPS patients was blocked by cathepsin B inhibitor CA074-Me,^7^ and that the cell death induced by transient expression of CAPS-associated mutant NLRP3 was blocked by CA074-Me and caspase inhibitors.^9^ The cell death caused by CAPS-associated NLRP3 mutants in the Tet-on system (Figure 3a) was attenuated by both CA074-Me and pan-caspase inhibitor Z-VAD-fmk (Figure 3a). These data indicate that loss of viability was the result of programmed cell death with no requirements for LPS treatment or exogenous DNA transfection.

Specific caspase inhibitors, however, did not block cell death (Figure 3b) nor did simultaneous addition of multiple specific caspase inhibitors (data not shown). These results suggest that loss of viability was not dependent on caspase activities. The other pan-caspase inhibitors, Boc-D-fmk, ac-VAD-cho and Q-VD-OPh also failed to block cell death even at higher concentrations (Figure 3c and Supplementary Figure S4a-b). The results suggest that Z-VAD-fmk inhibited cell death presumably due to its ‘off-target' effects.

### Z-VAD-fmk and CA074-Me inhibited necrotic cell death at different steps

Without doxycycline treatment, we observed widely distributed ASC in the cells stably expressing mCherry-tagged ASC. Those cells were unsusceptible to Yo-Pro-1 staining. However, when we expressed CAPS-associated mutant NLRP3 by doxycycline, the diffused presence of ASC changed to a speckle pattern and then became ‘green' (positive) when stained with Yo-Pro-1, reflecting cell membrane damage (Supplementary Figure S4c).

**Figure 4:**
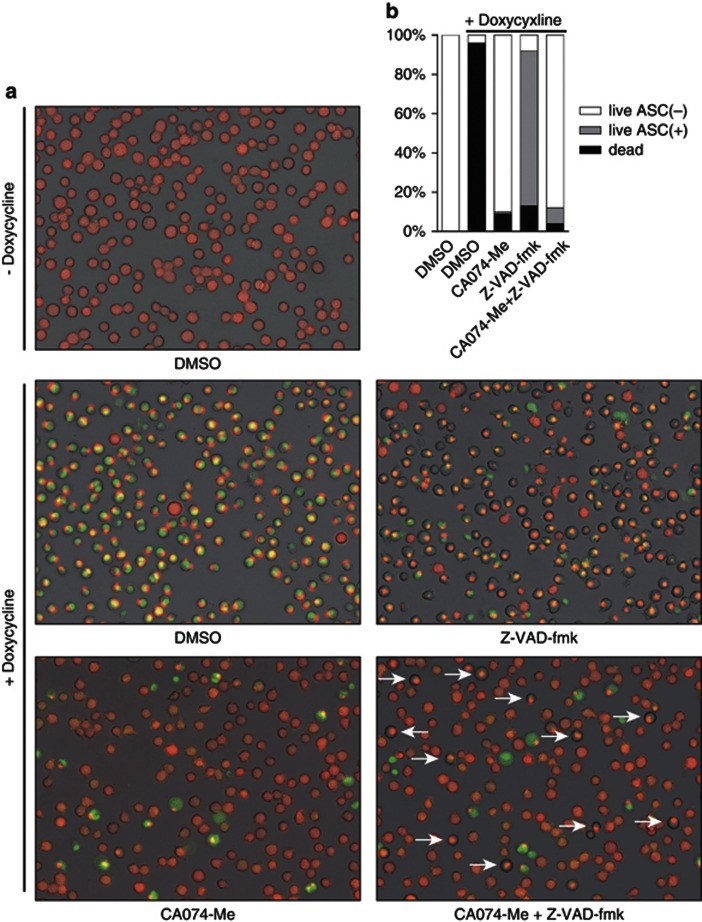
Z-VAD-fmk and CA074-Me inhibited necrotic cell death at different steps. (**a** and **b**) D301N-NLRP3-Tet-on-MC/9 cells stably expressing ASC-mCherry-pMX-IN were treated with 1 *μ*g/ml doxycycline for 12 h in addition to 50 *μ*M CA074-Me or 50 *μ*M Z-VAD-fmk. Cells were stained with 0.1 *μ*M Yo-Pro-1 for 5 min and cell morphologies were examined under a fluorescent microscope. The number of cells containing ASC speckling among Yo-Pro-1 negative live cells was counted; enumeration included over 100 cells based on pictures taken with a Carl ZEISS Axio Observer D1 for three independent trials

After mutant NLRP3 expression by doxycycline under DMSO treatment as vehicle, most cells converted the distribution pattern of ASC to speckle pattern and became positive for Yo-Pro-1 (Figure 4a, upper and middle left panels, and Figure 4b). Expression of mutant NLRP3 under Z-VAD-fmk treatment also resulted in the formation of ASC speckle, but importantly, most cells stayed negative for Yo-Pro-1 (Figure 4a, middle right panel, and Figure 4b), indicating that those cells could avoid the cell death even after ASC speckle formation. In contrast, when treated with CA074-Me, most cells retained the diffused appearance of ASC and negative for Yo-Pro-1 (Figure 4a, lower left panel, and Figure 4b), suggesting that CA074-Me inhibited the cell death before ASC oligomerization step.

In order to further confirm the processes those inhibitors blocked in the cell death pathway, we treated the cells simultaneously with CA074-Me and Z-VAD-fmk (Figure 4a, lower right panel), and observed that most cells showed the diffused presence of ASC, as did CA074-Me-treated cells. Moreover, some cells escaped cell death with ASC speckling (Figure 4a, lower right panel, indicated by arrows), leading to an overall decrease of dead cells in number compared with the condition treated with CA074-Me alone (Figure 4b). This result indicated that some cells that were not blocked by CA074-Me were rescued by Z-VAD-fmk even after ASC oligomerization, supporting the idea that CA074-Me worked on a step prior to both of ASC oligomerization and the process that was susceptible to Z-VAD-fmk.

### ASC was required in NLRP3-induced necrotic cell death, whereas caspase-1 was not

**Figure 5:**
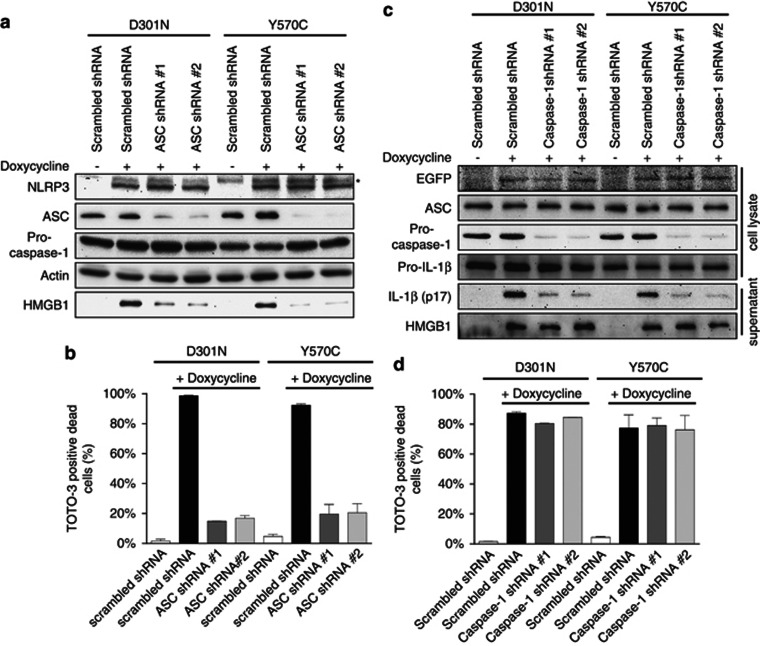
ASC was required for necrotic cell death induced by CAPS-associated mutant NLRP3, whereas caspase-1 was not. (**a**) D301N and Y570C-NLRP3-Tet-on-MC/9 cells were transduced with scrambled or *Asc*-targeting shRNA lentiviral vector and selected by puromycin for 1 week. Cells (1 × 10^6^) in 1 ml of medium were treated with 1 *μ*g/ml doxycycline. Cells were collected 3 h later while supernatants were harvested 24 h after doxycycline treatment. Immunoblotting of NLRP3, ASC, caspase-1, actin and HMGB1 was performed on the cells or the supernatant. The asterisk indicates non-specific band. (**b**) D301N and Y570C-NLRP3-Tet-on-MC/9 cells transduced with scrambled or *Asc*-targeting shRNA lentiviral vector were treated with 1 *μ*g/ml doxycycline for 12 h. Cells were stained with 100 nM TOTO-3 for 5 min, and TOTO-3-positive dead cells were counted by flow cytometry. (**c**) D301N and Y570C-NLRP3-Tet-on-MC/9 cells were transduced with scrambled or *Casp1*-targeting shRNA lentiviral vector and selected by puromycin for 1 week. Cells were transfected with the pro-IL-1*β*-pMX-IP retroviral vector and incubated for 48 h. Cells (1 × 10^6^) in 1 ml of medium were treated with 1 *μ*g/ml doxycycline. Cells were collected 3 h later while supernatants were harvested 12 h after doxycycline treatment. Immunoblotting of EGFP, ASC, caspase-1, IL-1*β* and HMGB1 was performed on the cells or the supernatant. (**d**) D301N or Y570C-NLRP3-Tet-on-MC/9 cells (in which caspase-1 was knocked down) were treated with 1 *μ*g/ml doxycycline for 12 h. Cells were stained with 100 nM TOTO-3 for 5 min, and TOTO-3-positive dead cells were counted by flow cytometry

In order to investigate whether ASC oligomerization was only an accessory event or a critical process for programmed cell death, we knocked down ASC in NLRP3-Tet-on-MC/9 cells. This led to decreases of both HMGB1 release and TOTO-3-positive dead cells upon expression of D301N and Y570C (Figures 5a and b), even though NLRP3 was expressed at similar levels in scrambled-small hairpin RNA (shRNA)- and *Asc*-shRNA-expressing NLRP3-Tet-on-MC/9 cells (Figure 5a). Thus, cell death induced by NLRP3 activation required ASC.

The caspase-1 inhibitor did not block NLRP3-induced cell death (Figure 3b). That result indicated that the activity of caspase-1 was not required for the cell death. We further investigated the possible requirement for caspase-1 in cell death by using shRNA that targeted caspase-1. Knocking down caspase-1 did not block cell death induced by expressing CAPS-associated mutant NLRP3 (Figures 5c and d). These results likely indicate that caspase-1 was not involved in cell death and support the previous report that macrophages derived from caspase-1 knockout mice also showed NLRP3/ASC-dependent cell death and exacerbated the inflammatory response induced by *Shigella flexneri*.^6^

### NLRP3-mediated necrotic cell death resulted in a neutrophilic inflammatory response without IL-1*β*

CAPS patients are known to have neutrophil-rich urticarial rashes.^20^ In order to investigate if the necrotic cell death caused by activated NLRP3 induced a neutrophilic inflammatory response, we performed neutrophil infiltration assays using an air-pouch model. NLRP3-Tet-on-MC/9 cells were injected and incubated in the air-pouch and all the cells in the pouch were collected for flow cytometric analysis. WT-NLRP3-Tet-on-MC/9 cells increased the level of NLRP3 expression upon doxycycline administration (Supplementary Figure S5a, middle panels). Importantly, the injected WT-NLRP3-Tet-on-MC/9 cells retained viability even after doxycycline administration (Supplementary Figure S5a, upper panels, and Supplementary Figure S5b). In contrast, mutant NLRP3-Tet-on-MC/9 cells disappeared after doxycycline administration, presumably due to necrotic cell death (Supplementary Figure S5a, upper panels, and Supplementary Figure S5b).

**Figure 6:**
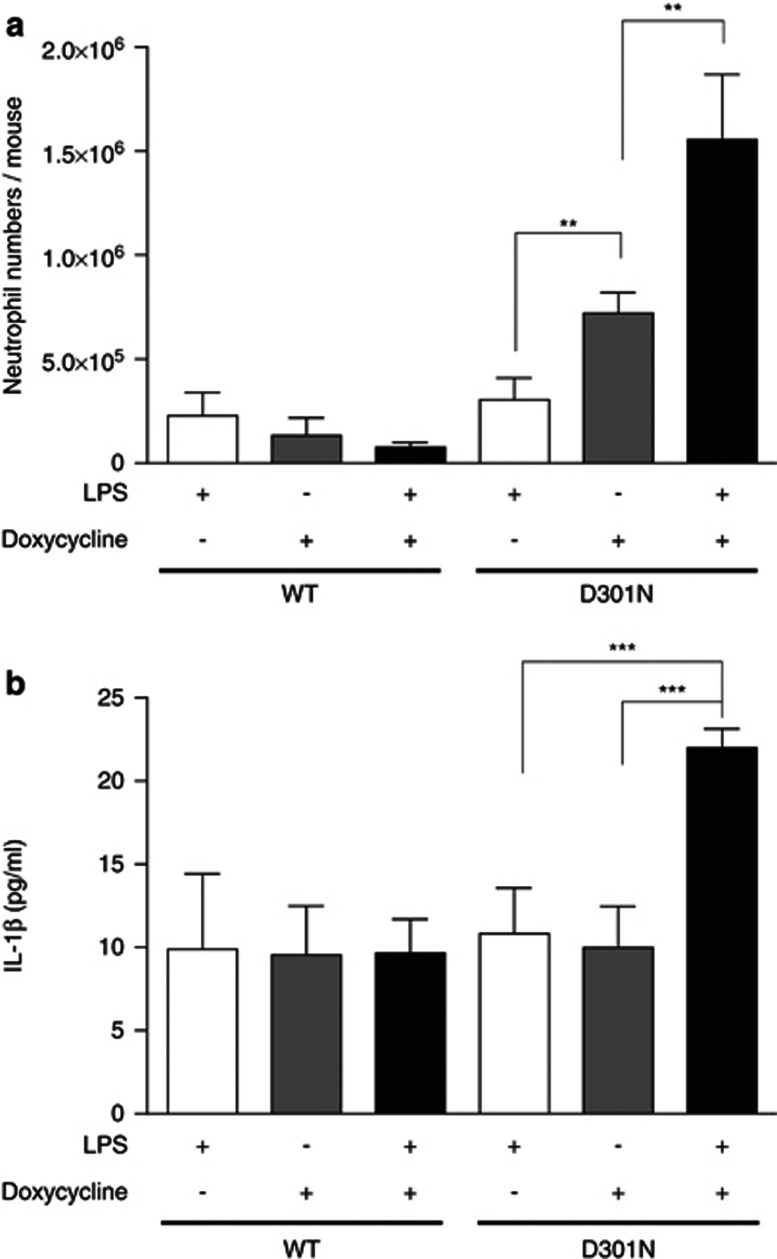
Necrotic cell death recruited neutrophils without IL-1*β*. (**a**) WT or D301N-NLRP3-Tet-on-MC/9 cells (1 × 10^7^) pretreated with 0.5 *μ*g/ml LPS for 2 h were injected into an air-pouch located on the backs of mice. Some mice were given doxycycline-containing drinking water 24 h before cell injection. After 16 h, the cells in the pouch were harvested by injecting 1 ml PBS and stained with Alexa647-Gr-1 and PE-F4/80 antibodies for flow cytometric analysis. Cells located in the gate A (Supplementary Fig. S5a), other than MC/9 (gate B) or dead cells, were analyzed for Alexa647-Gr-1 and PE-F4/80 axes. (**b**) WT or D301N-NLRP3-Tet-on-MC/9 cells (1 × 10^7^), pretreated with 0.5 *μ*g/ml LPS for 2 h, were injected into an air-pouch. Some mice were given doxycycline-containing drinking water 24 h before cell injection. After 8 h, the lavage fluid in the air-pouch was harvested by injecting 300 *μl* PBS and IL-1*β* ELISA was performed. All results are representative of at least three separate experiments (*n*=5 mice per condition). **, *P*<0.01; and ***, *P*<0.001

Pretreatment with LPS induces the expression of pro-IL-1*β*. Consequently, mature IL-1*β* is secreted from LPS-pretreated cells when expressing CAPS-associated mutant NLRP3. Mature IL-1*β* secretion combined with necrotic cell death in the air-pouch resulted in neutrophil infiltration (Figures 6a and b and Supplementary Figure S5a, lower panels). In contrast, neutrophils did not increase in the air-pouch injected with WT-NLRP3-Tet-on-MC/9 cells, unaccompanied by IL-1*β* release (Figure 6b). Necrotic cell death without IL-1*β* secretion (which can be achieved in this system by administering doxycycline without LPS pretreatment) also induced neutrophil infiltration, though fewer in number relative to that induced by necrotic cell death with IL-1*β* (Figure 6a). Such data indicate that necrotic cell death in itself can cause and exacerbate the neutrophilic inflammatory response.

## Discussion

In this study, we showed that expression of CAPS-associated mutant NLRP3 itself induced programmed necrotic cell death independently of IL-1*β* processing and that cell death induced by activated NLRP3 exacerbated the inflammatory response in addition to IL-1*β*. We clarified the mechanism of NLRP3-mediated cell death by using a CAPS-associated mutant NLRP3, thereby avoiding exposure to a pathogen, LPS stimulation or cell damage caused by second signals. Our study further supports previous data showing that transient expression of CAPS-associated mutant NLRP3-induced necrotic cell death,^9^ and that LPS, accompanied with mutant NLRP3 expression, induced necrotic cell death in monocytes isolated from CAPS patients.^6, 7, 8^

The characteristic programmed necrosis regulated by NLRP3 has been termed pyronecrosis.^6^ In pyronecrosis, cell lysis and HMGB1 release occurred after ASC speckle formation and cell death was inhibited by Z-VAD-fmk. This report led us to compare pyronecrosis and pyroptosis, the latter being caspase-1-dependent programmed necrosis in response to intracellular bacteria such as *Salmonella*.^21, 22^ Pyroptosis requires caspase-1 activity and is inhibited by caspase-1 inhibitor or pan-caspase inhibitor Z-VAD-fmk.^23, 24, 25^ Our data, however, demonstrated that a caspase-1 inhibitor did not inhibit pyronecrosis, cell death caused by CAPS-associated mutant NLRP3 expression. Moreover, knocking down caspase-1 in our study did not prevent cell death, supporting the previous reports that macrophages derived from caspase-1 knockout mice also showed NLRP3-mediated pyronecrosis induced by *Shigella*.^6^

Most pathogens induce not only NLRP3 but also pro-IL-1*β* expression. Therefore, the effect of NLRP3-mediated pyronecrosis is the combined outcome of cell death and IL-1*β*. Here, we first time show that NLRP3-mediated pyronecrosis recruits neutrophils even in the absence of IL-1*β* and it may contribute to the clearance of pathogens. The result that NLRP3-mediated cell death might possess the ability to evoke inflammation even in the absence of IL-1*β* is reasonable, and hence pyronecrosis does not require the activity of caspase-1 that cleaves IL-1*β* to its mature form.

Cathepsin B is a cysteine protease that is normally located in lysosomes and degrades various proteins in an acidic environment.^26^ Cathepsin B can be lethal if released from the lysosomal compartment. Cathepsin B inhibitor CA074-Me abrogated NLRP3-dependent pyronecrois so that several groups, including us, deduced that cathepsin B was involved in pyronecrosis after inflammasome formation.^9, 27^ However, if CA074-Me could inhibit the cell death at the step after inflammasome formation, the rescued live cells by CA074-Me must bear ASC speckle, which was not occurred in our experiment. This observation may suggest that the hypothesis that cathepsin B is involved in pyronecrosis after inflammasome formation is incorrect, and that cathepsin B has some role in the process prior to inflammasome formation. This idea is also supported from the finding that pyroptosis, which is ASC-mediated cell death, was not blocked by cathepsin B inhibitor.^28^

Cathepsin B released from ruptured lysosomes after phagocytosis of large particles such as silica or *β*-amyloid is thought to cleave an unidentified substrate and trigger activation of the inflammasome.^29, 30^ However, when cells expressed the CAPS-associated mutant NLRP3 with the Tet-on system, a cathepsin B inhibitor blocked both cell death and mature IL-1*β* release (Supplementary Figure S5c), even though a formation of large particles and lysosomal rupture was not involved (data not shown). Consider also that LPS-induced death of human monocytes carrying the NLRP3 mutation (which cannot phagocytize large particles) was also blocked by a cathepsin B inhibitor.^7^ As CA074-Me inhibited NLRP3-mediated cell death even without a formation of large particles and lysosomal rupture, careful attention should be paid to the deduced involvement of cathepsin B in inflammasome formation. Thus, the theory might require re-evaluation that phagocytosis of large particles induces lysosome rupture and cytosolic activation of cathepsin B, resulting in NLRP3 activation. Additional information regarding how inhibitors such as CA074-Me and Z-VAD-fmk suppress cell death might provide clues, clarifying the mechanism of inflammasome activation and NLRP3-mediated necrotic programmed cell death.

In this study, we demonstrated that expression of CAPS-associated mutant NLRP3 itself induced programmed necrotic cell death downstream of ASC oligomerization, independent of IL-1*β* processing. This type of cell death may have wide significance in immune responses because it is mediated by NLRP3, a protein that senses not only pathogens but also danger-associated signals, and presumably contributes to neutrophil infiltration in urticarial rashes in CAPS or other diseases such as gout in which NLRP3 is proposed to contribute to the pathogenesis.

## Materials and Methods

### Plasmids

The expression plasmids for mouse NLRP3-EGFP, its CAPS-associated-mutants (namely R258W, D301N and Y570C) and ASC were described previously.^19^ In order to establish the Tet-on system, NLRP3-EGFP and its mutants were inserted into pRetroX-Tight-Pur or pRetroX-Tight-Hyg retrovirus vectors (Clontech, Mountain View, CA, USA). The following primers were used to amplify the pro-lL-1*β* transcript: forward primer, 5′-GCGCTCGAGGCAGCTATGGCAACTGTTCCTG-3′, and reverse primer, 5′-CGCGCGGCCGCTTAGGAAGACACGGATTCCATGG-3′. ASC was fused with mCherry fluorescent protein (Clontech). The pro-IL-1*β* transcript and ASC-mCherry were cloned into pMX-IRES-puro (pMX-IP) vector (provided by Dr. T Kitamura, University of Tokyo, Tokyo, Japan) or pMX-IRES-human nerve growth factor receptor p75 (pMX-IN) vector (provided by Drs. A Onodera and T Nakayama, Chiba University, Chiba, Japan).

### Generation of cell lines

Mouse mast cell line MC/9 cells and mouse monocyte cell line J774A.1 cells were maintained in DMEM medium with 10% FBS. In order to establish NLRP3-Tet-on-MC/9 and NLRP3-Tet-on-J774A.1 cells by the Tet-on system, viral transduction was performed as described previously.^19^ MC/9 and J774A.1 cells were incubated with retroviral supernatants for 15 h with 4 *μ*g/ml polybrene (Sigma-Aldrich, St Louis, MO, USA), and selected with G418 (3 mg/ml), puromycin (3 *μ*g/ml) or hygromycin (50 *μ*g/ml), depending on the selection markers. Cells transfected with expression vector containing human nerve growth factor receptor were enriched twice by MACS with anti-human NGFR (C40-1457; BD Pharmingen, San Jose, CA, USA).

### Western blot Assays

Cells were washed in PBS and lysed in ice-cold lysis buffer M-PER (Thermo Fisher, Waltham, MA, USA) supplemented with protease inhibitor cocktails. Lysates and supernatants were centrifuged for 5 min and the supernatants were boiled for 5 min in sample buffer. After SDS-PAGE, immunoblots were processed using antibodies against EGFP (Clontech), NLRP3 (Cryo-2; AdipoGen, San Diego, CA, USA), ASC (a gift from Dr. J Masumoto, Shinshu University, Nagano, Japan), caspase-1 (sc-514; Santa Cruz, Dallas, TX, USA), IL-1*β* (AB-401; R&D Systems, Minneapolis, MN, USA), HMGB1 (14-9900; eBioscience, San Diego, CA, USA), actin (sc-8432; Santa Cruz), caspase-3 (9661; Cell Signaling, Danvers, MA, USA) or PARP (9542; Cell Signaling).

### Reagents

The pan-caspase inhibitors Z-VAD-fmk (R&D Systems), ac-VAD-cho (MERCK, Whitehouse Station, NJ, USA), Q-VD-OPh and Boc-D-fmk (BioVision, Milpitas, CA, USA), and specific caspase inhibitors Z-YVAD-fmk (MERCK), Z-VDVAD-fmk, Z-LEVD-fmk and Z-ATAD-fmk (MBL, Nagoya, Japan), Z-YVAD-cho, Z-DEVD-fmk, Z-IETD-fmk and Z-LEHD-fmk (R&D Systems), Z-FA-fmk (BD Pharmingen) were purchased. Cathepsin B inhibitor CA074-Me was obtained from MERCK. Ultra pure LPS was from InvivoGen, San Diego, CA, USA. ATP was from Sigma-Aldrich. Doxycycline and Tet system-approved FBS were obtained from Clontech.

### Flow cytometry

Expression levels of EGFP-tagged proteins were assessed by flow cytometry (Canto II, BD, Franklin Lakes, NJ, USA). Dead cells were analyzed using TOTO-3 (Invitrogen, San Diego, CA, USA) staining.

### Apoptosis and necrosis assays

The mode of cell death, namely apoptosis or necrosis, was determined primarily by the morphology of the dying cells under microscopic observation. The proportions of apoptotic and necrotic cells were also determined by flow cytometry^9^ after staining with Alexa 647-annexin-V (BioLegend, San Diego, CA, USA) and 7-AAD (BD Pharmingen).

### ELISA assays

The amount of IL-1*β* was determined using an OptELISA kit (eBioscience), according to the manufacturer's protocol.

### LDH assays

LDH activities in the culture supernatants were measured using a CytoTox 96 assay kit (Promega, Madison, WI, USA).

### Electron microscopy

Cells were fixed in 2% glutaraldehyde in phosphate buffer. Routine procedures for observation by electron microscopy were performed by Filgen Inc (Nagoya, Japan).

### Knockdown of ASC and caspase-1

ASC or caspase-1 expression was knocked down using shRNA inserted into a lentiviral pLKO.1 puro vector (Sigma-Aldrich). MC/9 cells stably expressing Tet-on inducible NLRP3 were exposed to *Asc*-targeting, *Casp1*-targeting or control shRNA lentivirus and selected by puromycin.

### Fluorescent microscopy

Cells were observed with a Carl Zeiss Axio Observer microscope or Olympus FV10i. In some experiments, cells were stained with 0.1 *μ*M Yo-Pro-1 (Invitrogen) for 5 min to distinguish necrotic dead cells from living cells.

### Air-pouch model

The Chiba University Animal Care and Use Committee approved the animal procedures used in this study. Air pouches were established in 4-week-old male C57BL/6 mice by injecting 3 and 1.5 ml of sterile air at day 0 and day 3, respectively, under the dorsal skin of mice.^31^ At day 5, mice were administered with 2 mg/ml doxycycline in drinking water. At day 6, NLRP3-Tet-on-MC/9 cells were pretreated with 0.5 *μ*g/ml LPS for 2 h, washed with sterile PBS three times, incubated in culture medium for 4 h and washed three times with sterile PBS to eliminate the effect of tumor necrosis factor-*α*, mainly released 3 h after LPS stimulation (data not shown). After these pretreatments, mice were injected with 1 × 10^7^ NLRP3-Tet-on-MC/9 cells in 1 ml PBS. At day 7, the pouches were washed with 1 ml of PBS and the lavage fluid and cells were harvested for ELISA and FACS analysis with PE-anti-mouse F4/80 and Alexa647-anti-mouse Gr-1 (Ly-6G) antibodies from BioLegend.

## Abbreviations

ASC: apoptosis-associated speck-like protein containing a CARD
CAPS: cryopyrin-associated periodic syndrome
HMGB1: high-mobility group box 1
IL: interleukin
LPS: lipopolysaccharide
NLR: NOD-like receptor
NLRP3: NLR family pyrin domain containing 3
shRNA: small hairpin RNA

## References

[bib1] Tschopp J Schroder K NLRP3 inflammasome activation: The convergence of multiple signalling pathways on ROS production Nat Rev Immunol 2010 10 210 215 20168318 10.1038/nri2725

[bib2] Meylan E Tschopp J Karin M Intracellular pattern recognition receptors in the host response Nature 2006 442 39 44 16823444 10.1038/nature04946

[bib3] Jones DA Takemoto D Plant innate immunity - direct and indirect recognition of general and specific pathogen-associated molecules Curr Opin Immunol 2004 16 48 62 14734110 10.1016/j.coi.2003.11.016

[bib4] Chisholm ST Coaker G Day B Staskawicz BJ Host-microbe interactions: shaping the evolution of the plant immune response Cell 2006 124 803 814 16497589 10.1016/j.cell.2006.02.008

[bib5] Hoffman HM Mueller JL Broide DH Wanderer AA Kolodner RD Mutation of a new gene encoding a putative pyrin-like protein causes familial cold autoinflammatory syndrome and Muckle-Wells syndrome Nat Genet 2001 29 301 305 11687797 10.1038/ng756 PMC4322000

[bib6] Willingham SB Bergstralh DT O'Connor W Morrison AC Taxman DJ Duncan JA Microbial pathogen-induced necrotic cell death mediated by the inflammasome components CIAS1/cryopyrin/NLRP3 and ASC Cell Host Microbe 2007 2 147 159 18005730 10.1016/j.chom.2007.07.009 PMC2083260

[bib7] Saito M Nishikomori R Kambe N Fujisawa A Tanizaki H Takeichi K Disease-associated CIAS1 mutations induce monocyte death, revealing low-level mosaicism in mutation-negative cryopyrin-associated periodic syndrome patients Blood 2008 111 2132 2141 18063752 10.1182/blood-2007-06-094201

[bib8] Reddy S Jia S Geoffrey R Lorier R Suchi M Broeckel U An autoinflammatory disease due to homozygous deletion of the IL1RN locus N Engl J Med 2009 360 2438 2444 19494219 10.1056/NEJMoa0809568 PMC2803085

[bib9] Fujisawa A Kambe N Saito M Nishikomori R Tanizaki H Kanazawa N Disease-associated mutations in CIAS1 induce cathepsin B-dependent rapid cell death of human THP-1 monocytic cells Blood 2007 109 2903 2911 17164343 10.1182/blood-2006-07-033597

[bib10] Gilroy EM Hein I van der Hoorn R Boevink PC Venter E McLellan H Involvement of cathepsin B in the plant disease resistance hypersensitive response Plant J 2007 52 1 13 17697096 10.1111/j.1365-313X.2007.03226.x

[bib11] Rathinam VA Jiang Z Waggoner SN Sharma S Cole LE Waggoner L The AIM2 inflammasome is essential for host defense against cytosolic bacteria and DNA viruses Nat Immunol 2010 11 395 402 20351692 10.1038/ni.1864 PMC2887480

[bib12] Fernandes-Alnemri T Yu JW Juliana C Solorzano L Kang S Wu J The AIM2 inflammasome is critical for innate immunity to Francisella tularensis Nat Immunol 2010 11 385 393 20351693 10.1038/ni.1859 PMC3111085

[bib13] Jones JW Kayagaki N Broz P Henry T Newton K O'Rourke K Absent in melanoma 2 is required for innate immune recognition of Francisella tularensis Proc Natl Acad Sci USA 2010 107 9771 9776 20457908 10.1073/pnas.1003738107 PMC2906881

[bib14] Nakahira K Haspel JA Rathinam VA Lee SJ Dolinay T Lam HC Autophagy proteins regulate innate immune responses by inhibiting the release of mitochondrial DNA mediated by the NALP3 inflammasome Nat Immunol 2011 12 222 230 21151103 10.1038/ni.1980 PMC3079381

[bib15] Hanley PJ Kronlage M Kirschning C del Rey A Di Virgilio F Leipziger J Transient P2 × 7 receptor activation triggers macrophage death independent of Toll-like receptors 2 and 4, caspase-1, and pannexin-1 proteins J Biol Chem 2012 287 10650 10663 22235111 10.1074/jbc.M111.332676 PMC3323034

[bib16] Hentze H Lin XY Choi MS Porter AG Critical role for cathepsin B in mediating caspase-1-dependent interleukin-18 maturation and caspase-1-independent necrosis triggered by the microbial toxin nigericin Cell Death Differ 2003 10 956 968 12934070 10.1038/sj.cdd.4401264

[bib17] Scaffidi P Misteli T Bianchi ME Release of chromatin protein HMGB1 by necrotic cells triggers inflammation Nature 2002 418 191 195 12110890 10.1038/nature00858

[bib18] Agostini L Martinon F Burns K McDermott MF Hawkins PN Tschopp J NALP3 forms an IL-1beta-processing inflammasome with increased activity in Muckle-Wells autoinflammatory disorder Immunity 2004 20 319 325 15030775 10.1016/s1074-7613(04)00046-9

[bib19] Nakamura Y Kambe N Saito M Nishikomori R Kim YG Murakami M Mast cells mediate neutrophil recruitment and vascular leakage through the NLRP3 inflammasome in histamine-independent urticaria J Exp Med 2009 206 1037 1046 19364881 10.1084/jem.20082179 PMC2715029

[bib20] Shinkai K McCalmont TH Leslie KS Cryopyrin-associated periodic syndromes and autoinflammation Clin Exp Dermatol 2008 33 1 9 17927785 10.1111/j.1365-2230.2007.02540.x

[bib21] Fernandes-Alnemri T Wu J Yu JW Datta P Miller B Jankowski W The pyroptosome: a supramolecular assembly of ASC dimers mediating inflammatory cell death via caspase-1 activation Cell Death Differ 2007 14 1590 1604 17599095 10.1038/sj.cdd.4402194 PMC3345951

[bib22] Monack DM Detweiler CS Falkow S Salmonella pathogenicity island 2-dependent macrophage death is mediated in part by the host cysteine protease caspase-1 Cell Microbiol 2001 3 825 837 11736994 10.1046/j.1462-5822.2001.00162.x

[bib23] Brennan MA Cookson BT Salmonella induces macrophage death by caspase-1-dependent necrosis Mol Microbiol 2000 38 31 40 11029688 10.1046/j.1365-2958.2000.02103.x

[bib24] Bergsbaken T Cookson BT Macrophage activation redirects yersinia-infected host cell death from apoptosis to caspase-1-dependent pyroptosis PLoS Pathog 2007 3 e161 17983266 10.1371/journal.ppat.0030161 PMC2048529

[bib25] Reisetter AC Stebounova LV Baltrusaitis J Powers L Gupta A Grassian VH Induction of inflammasome-dependent pyroptosis by carbon black nanoparticles J Biol Chem 2011 286 21844 21852 21525001 10.1074/jbc.M111.238519 PMC3122239

[bib26] Turk V Stoka V Vasiljeva O Renko M Sun T Turk B Cysteine cathepsins: from structure, function and regulation to new frontiers Biochim Biophys Acta 2012 1824 68 88 22024571 10.1016/j.bbapap.2011.10.002 PMC7105208

[bib27] Duncan JA Gao X Huang MT O'Connor BP Thomas CE Willingham SB Neisseria gonorrhoeae activates the proteinase cathepsin B to mediate the signaling activities of the NLRP3 and ASC-containing inflammasome J Immunol 2009 182 6460 6469 19414800 10.4049/jimmunol.0802696 PMC2722440

[bib28] Lamkanfi M Sarkar A Vande Walle L Vitari AC Amer AO Wewers MD Inflammasome-dependent release of the alarmin HMGB1 in endotoxemia J Immunol 2010 185 4385 4392 20802146 10.4049/jimmunol.1000803 PMC3428148

[bib29] Hornung V Bauernfeind F Halle A Samstad EO Kono H Rock KL Silica crystals and aluminum salts activate the NALP3 inflammasome through phagosomal destabilization Nat Immunol 2008 9 847 856 18604214 10.1038/ni.1631 PMC2834784

[bib30] Halle A Hornung V Petzold GC Stewart CR Monks BG Reinheckel T The NALP3 inflammasome is involved in the innate immune response to amyloid-beta Nat Immunol 2008 9 857 865 18604209 10.1038/ni.1636 PMC3101478

[bib31] Sin YM Sedgwick AD Chea EP Willoughby DA Mast cells in newly formed lining tissue during acute inflammation: a six day air pouch model in the mouse Ann Rheum Dis 1986 45 873 877 3789822 10.1136/ard.45.10.873 PMC1002011

