# Supplementary figures and images for: NLRP3 activation induces ASC-dependent programmed necrotic cell death, which leads to neutrophilic inflammation
Source: Cell Death Dis. 2013 May 23;4(5):e644–. doi: 10.1038/cddis.2013.169 (PMC3674376; doi:10.1038/cddis.2013.169)

Supplementary Figure S1

a

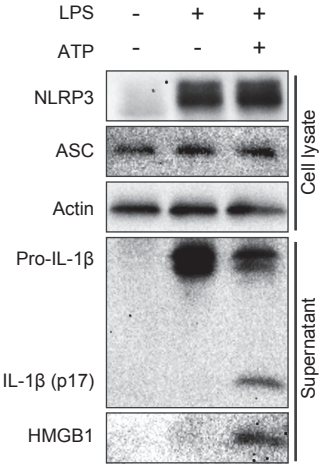

b

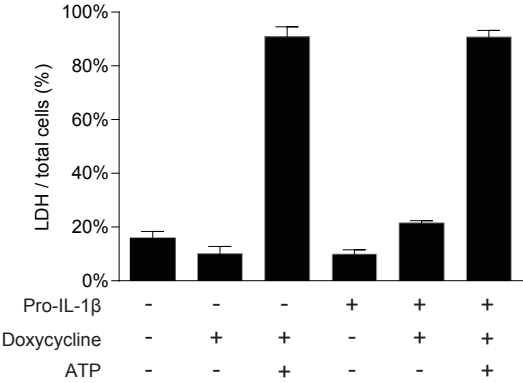

c

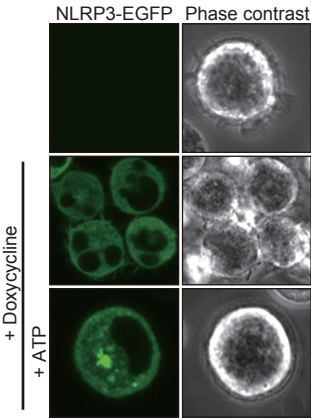

Supplement: Supplementary Figure S1 [file cddis2013169x1.pdf]

Supplementary Figure S2

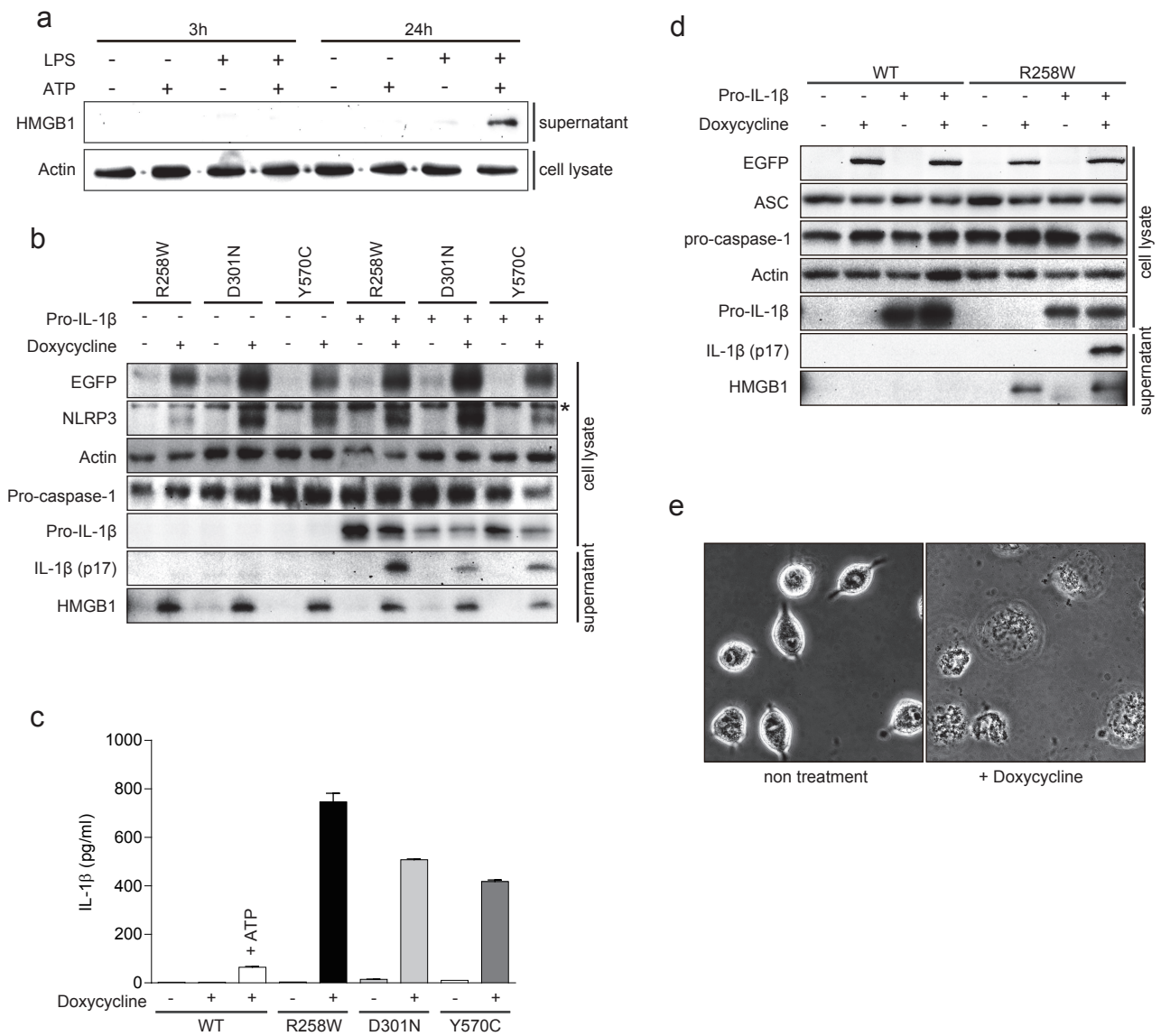

Supplement: Supplementary Figure S2 [file cddis2013169x2.pdf]

Supplementary Figure S3

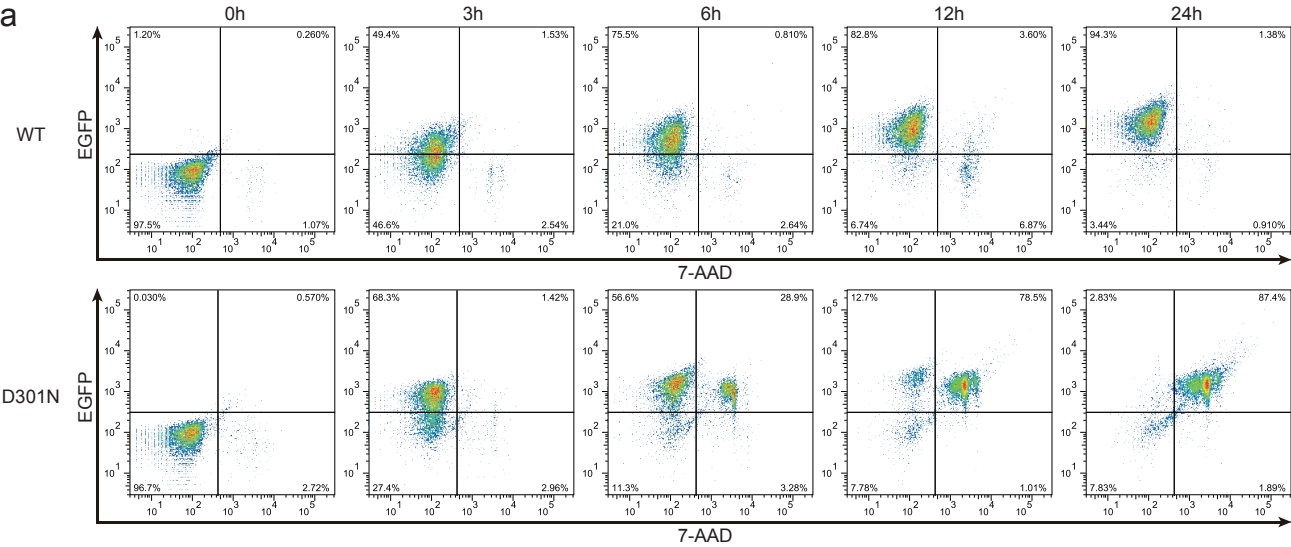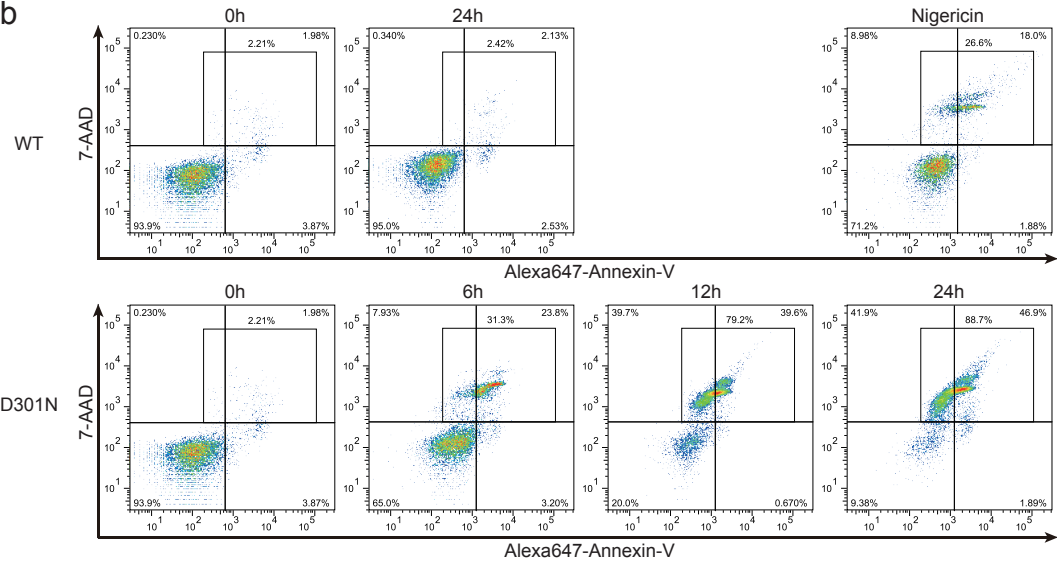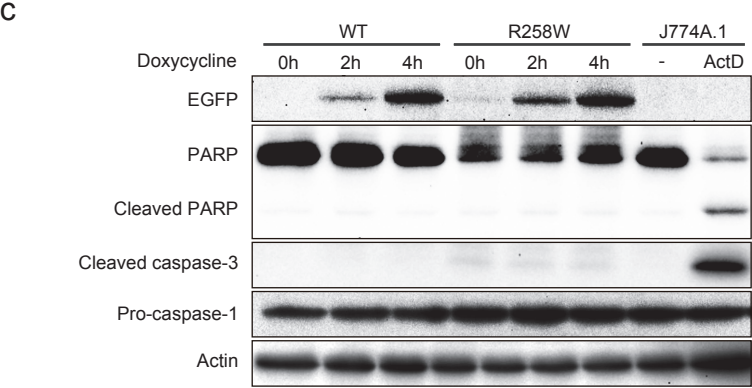

Supplement: Supplementary Figure S3 [file cddis2013169x3.pdf]

Supplementary Figure S4

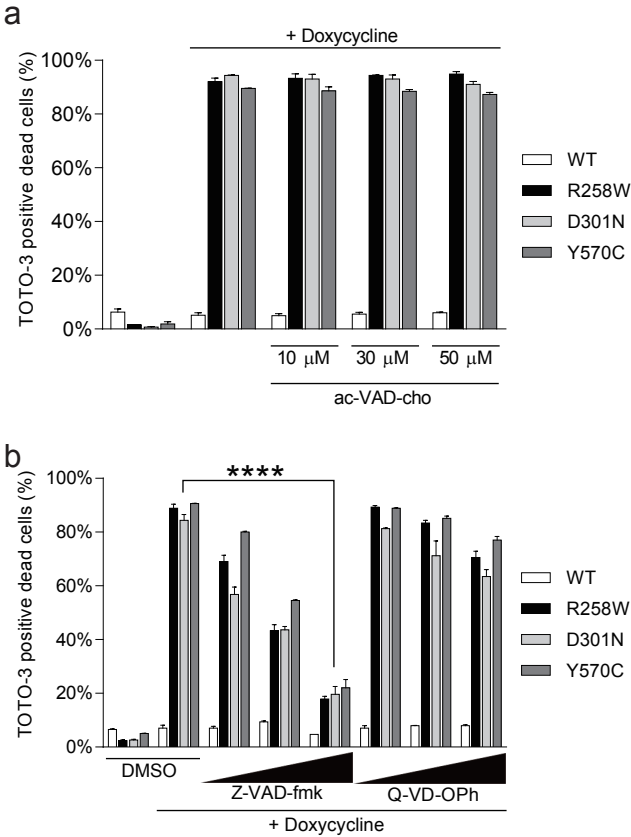

**C**

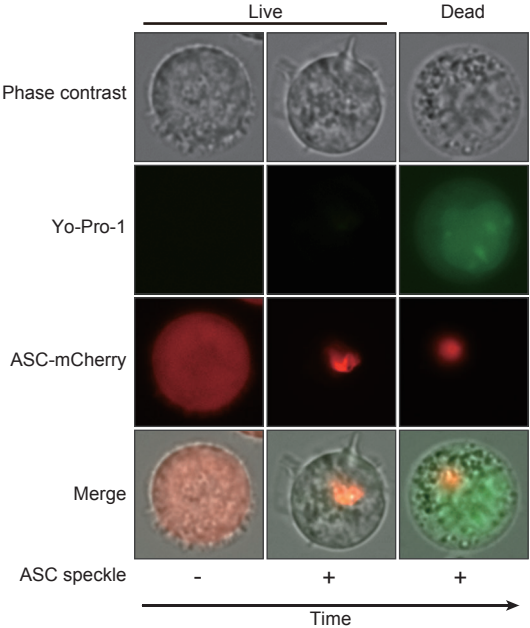

Supplement: Supplementary Figure S4 [file cddis2013169x4.pdf]

a

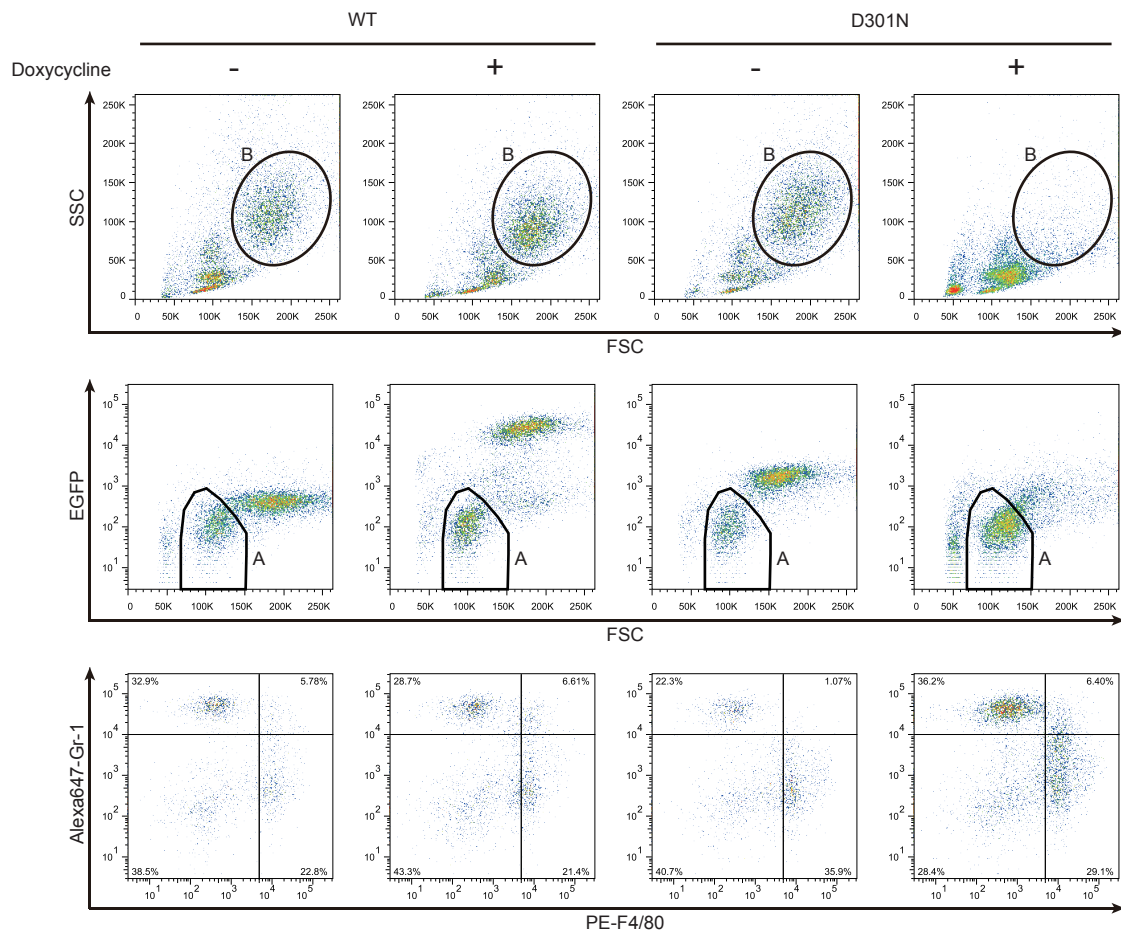

b

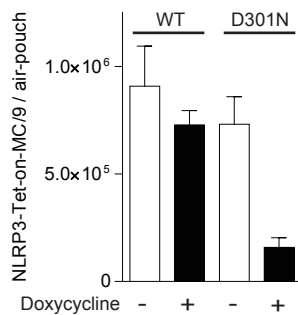

C

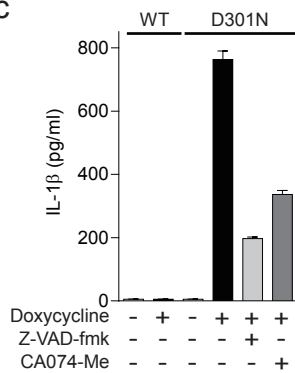

Supplement: Supplementary Figure S5 [file cddis2013169x5.pdf]
