# Supplementary material for: NLRP3 activation induces ASC-dependent programmed necrotic cell death, which leads to neutrophilic inflammation
Source: Cell Death Dis. 2013 May 23;4(5):e644–. doi: 10.1038/cddis.2013.169 (PMC3674376; doi:10.1038/cddis.2013.169)
Supplement: Supplementary Figure Legends [file cddis2013169x6.doc]

**Supplementary Figure S1.** WT-NLRP3 requires a second signal such as ATP to induce inflammasome formation and production of IL-1β. (**a**) LPS treatment induced NLRP3 and pro-IL-1β expression, but mature IL-1β was observed only after ATP treatment in the monocyte cell line J774A.1. J774A.1 cells (1 x 10^6^) were pretreated with 1 μg/mL LPS for 2 hours and stimulated with 5 mM ATP for 45 minutes. Cells and supernatants were harvested 45 minutes after ATP stimulation, except supernatants for HMGB1 immunoblotting were harvested 12 hours after ATP stimulation. (**b**) The supernatants harvested in Figure 1b were assessed by LDH assay. (**c**) WT-NLRP3-Tet-on-MC/9 cells were treated with 1 μg/mL doxycycline for 12 hours and the fluorescence of NLRP3-EGFP was observed in the cytoplasm. NLRP3-EGFP speckle was observed 30 minutes after 5 mM ATP stimulation. Confocal scanning was performed with an Olympus FV10i.

**Supplementary Figure S2.** Induction of CAPS-associated-NLRP3 mutants was sufficient for cell death without IL-1β. (**a**) MC/9 cells (1 x 10^6^) were pretreated with 0.5 μg/mL LPS for 2 hours and stimulated with 5 mM ATP for 3 or 24 hours. Cells and supernatants were harvested for HMGB1 and actin immunoblotting. (**b**) Mutant NLRP3-Tet-on-MC/9 cells were transfected with retroviral vector coding pro-IL-1β-pMX-IP and incubated for 48 hours. Cells (1 x 10^6^) were treated with 1 μg/mL doxycycline and cells and supernatants were harvested 3 hours later. Supernatants for HMGB1 immunoblotting were harvested 12 hours after doxycycline treatment. The asterisk indicates non-specific band. (**c**) WT and mutant NLRP3-Tet-on-J774A.1 cells were transfected with a retroviral vector coding pro-IL-1β-pMX-IP and incubated for 48 hours. NLRP3-Tet-on-J774A.1 cells (1 x 10^6^) were treated with 1 μg/mL doxycycline for 12 hours. The supernatants were harvested 12 hours later. WT-NLRP3-Tet-on-J774A.1 cells were stimulated with 5 mM ATP for 1 hour after doxycycline treatment. The supernatants were harvested for ELISA. (**d**) NLRP3-Tet-on-J774A.1 cells (1 x 10^6^) were transfected with retroviral vector coding pro-IL-1β-pMX-IP and incubated for 48 hours. Cells (1 x 10^6^) were treated with 1 μg/mL doxycycline for NLRP3 induction and cells were harvested 3 hours later. Supernatants were harvested 12 hours after doxycycline treatment. (**e**) Confocal scan images of cell death caused by the expression of R258W in Tet-on-J774A.1 cells were taken before and 12 hours after doxycycline treatment. Scanning was performed with an Olympus FV10i.

**Supplementary Figure S3.** Cell death induced by CAPS-associated-NLRP3 mutants was necrosis. (**a, b**) WT and D301N-NLRP3-Tet-on-MC/9s were treated with 1 μg/mL doxycycline and the signal intensities of EGFP, Alexa647-annexin-V, and 7-AAD staining were analyzed by flow cytometry. WT-NLRP3-Tet-on-MC/9 cells were incubated with 20 μg/mL nigericin for 6 hours as a necrosis control. (**c**) WT and R258W-NLRP3-Tet-on-J77A.1 cells (1 x 10^6^) were treated with 1 μg/mL doxycycline for the indicated time and the cells were harvested for immunoblotting of EGFP, PARP, cleaved caspase-3, caspase-1 and actin. J774A.1 cells were incubated with 100 nM actinomycin D for 6 hours as an apoptosis control.

**Supplementary Figure S4.** Pan-caspase inhibitor ac-VAD-cho or Q-VD-OPh did not inhibit cell death induced by CAPS-associated-mutant NLRP3. (**a, b**) WT and mutant NLRP3-Tet-on-MC/9 cells were treated with 1 μg/mL doxycycline for 12 hours in addition to the pan-caspase-inhibitor ac-VAD-cho (10/30/50 μM) or Q-VD-OPh (10/20/40 μM). Cells were stained with 100 nM TOTO-3 for 5 minutes and analyzed by flow cytometry. (**c**) D301N-NLRP3-Tet-on-MC/9 cells stably expressing ASC-mCherry-pMX-IN were treated with 1 μg/mL doxycycline. Cells were stained with 0.1 μM Yo-Pro-1 for 5 minutes and cell morphologies were examined under a fluorescent microscope. The pictures were taken with a Carl ZEISS Axio Observer D1 for three independent trials.

**Supplementary Figure S5.** NLRP3-mediated necrotic cell death resulted in a neutrophilic inflammatory response without IL-1β. (**a, b**) WT or D301N-NLRP3-Tet-on-MC/9 cells (1 x 10^7^), pretreated with 0.5 μg/mL LPS for 2 hours, were injected into an air-pouch located on the backs of mice. Some mice were provided with doxycycline-containing drinking water 24 hours before cell injection. After 16 hours, cells were harvested from the air-pouch and stained with Alexa647-Gr-1 and PE-F4/80 antibodies for flow cytometric analysis. Cells other than MC/9 or dead cells shown in gate A in FSC/EGFP axis (middle panels) are displayed as a function of Alexa647-Gr-1 and PE-F4/80 fluorescence (lower panels). The number of MC/9 cells shown in gate B in FSC/SSC axis (upper panels) is displayed in (b). (**c**) WT or D301N-NLRP3-Tet-on-MC/9 cells were transfected with pro-IL-1β-pMX-IP retroviral vector and incubated for 48 hours. NLRP3-Tet-on-MC/9 cells (1 x 10^6^) were treated with 1 μg/mL doxycycline in addition to 50 μM CA074Me or 10 μM Z-VAD-fmk for 12 hours. The supernatants were harvested for ELISA.
